# A Brief Introduction to Intelligent Point Cloud Processing, Sensing, and Understanding: Part II

**DOI:** 10.3390/s25051310

**Published:** 2025-02-21

**Authors:** Miaohui Wang, Sukun Tian

**Affiliations:** 1 Guangdong Key Laboratory of Intelligent Information Processing, College of Electronics and Information Engineering, Shenzhen University, Shenzhen 518052, China; 2 School of Stomatology, Peking University, Beijing 100081, China; sukhum169@bjmu.edu.cn

## 1. Introduction

Point cloud, which represents the three-dimensional (3D) digital world, is one of the fundamental data carriers in many emerging applications [1], including the autonomous driving, robotics, and geospatial fields. Advancements in sensor technologies have facilitated the acquisition of point clouds from diverse platforms [2], perspectives, and spectra. This progress has underscored the necessity to address the generation [3], processing [4], analysis [5], and quality evaluation [6] of point cloud data.

The demand for intelligent point cloud processing and analysis has surged across various industries [7]. For examples, accurate 3D models enhance design, construction, and maintenance in construction and architecture [8], improving project outcomes and reducing costs. Integrating point cloud data into physical object systems ensures more reliable digital representations of structures [9]. In autonomous driving, real-time point cloud processing is critical for vehicle perception [10], where LiDAR sensors help vehicles to detect and navigate their environment, ensuring safety and reliability. Additionally, industries such as manufacturing, cultural heritage preservation, and urban planning rely on point cloud data for quality control, digital restoration, and city modeling. The incorporation of artificial intelligence (AI) has revolutionized point cloud applications [11], where AI-driven technology helps to automate object recognition, segmentation, and classification, significantly improving efficiency and accuracy [12].

This Special Issue serves as a comprehensive collection of recent advances in point cloud processing across various sensors. A total of ten contributions from the Republic of Korea, rhe UK, the PR China, Belgium, and the USA have been ultimately accepted for publication. These contributions delve into diverse aspects of point clouds, including dataset establishment, registration, detection, performance evaluation, receptive field analysis, and plant feature extraction. We provide a brief introduction to each of the collected contributions in the following section.

## 2. Overview of Contributions

Contribution 1 introduces an innovative framework to enhance LiDAR-based datasets for advanced driver-assistance systems (ADASs), improving the representation of distant objects by generating synthetic object points from real point clouds. The proposed framework consists of three key modules: (1) position determination identifies optimal locations and orientations for synthetic objects, employing ground filtering to separate ground and non-ground points; selects candidate positions; resolves collisions; and determines object poses; (2) object generation converts LiDAR data from Cartesian to spherical coordinates to generate synthetic points, where a spherical point-tracking technique and a “point wall” method mitigate excessive data loss, preserving object shape fidelity; (3) synthetic annotation automatically labels object points with attributes such as position, size, pose, and occlusion, ensuring consistency with datasets such as KITTI-360. Experimental results show that integrating these synthetic objects into training datasets enhances the performance of 3D detection models.

Contribution 2 advances 3D data processing by introducing a point cloud-specific attention mechanism to enhance convolutional neural networks (CNNs). Traditional CNNs struggle with point clouds due to its unstructured and unordered nature. To address these issues, the authors propose a channel attention mechanism tailored for point clouds, integrating it into the ConvPoint benchmark. The resulted module enhances feature emphasis, improving the network’s focus on critical information. Experimental results show that incorporating the attention mechanism increases the mean intersection over union (mIoU) score, outperforming the base ConvPoint framework. This enhancement enables more effective processing of complex point clouds, with applications in autonomous driving, robotics, and virtual reality.

Contribution 3 proposes a novel algorithm for efficiently extracting phenotypic traits of rice plants using terrestrial laser scanning (TLS) data. It is noteworthy that traditional manual measurements are labor-intensive and time-consuming, so this study proposes an automated approach leveraging TLS data to extract key phenotypic features, including crown diameter, stem perimeter, plant height, surface area, volume, and projected leaf area. The extraction process employs several point cloud processing methods: (1) a neighborhood search algorithm calculates crown diameter and stem perimeter by establishing geometric relationships between point clouds; (2) an alpha-shape algorithm reconstructs the plant’s 3D surface to determine surface area and volume; (3) extended hierarchical density-based spatial clustering is used to group plant stem point clouds and obtain the tiller number. Based on these algorithms, the study enhances the efficiency and accuracy of phenotypic data collection, offering a valuable resource for rice breeding and growth monitoring.

Contribution 4 introduces a flexible and adaptive framework that enhances feature learning through the innovative use of receptive field space (RFS) and attention mechanisms. Since traditional methods rely on manually defined local neighborhoods, they may be inflexible and may fail to capture both local details and global dependencies. To address these problems, the authors propose constructing an RFS mechanism that extracts effective features across multiple receptive field ranges, allowing adaptive scale selection for each point. Moreover, they develop an RFS attention method, which dynamically adjusts the network’s focus across receptive field ranges, enhancing feature representation capability. This mechanism is integrated into a network architecture for point cloud classification and segmentation. Experimental results show that the proposed RFS effectively captures both local and global features, leading to improved 3D point cloud analysis.

Contribution 5 presents a comprehensive evaluation of six point cloud registration methods for aligning computer-aided design (CAD) models with real-world 3D scans. Unlike prior studies relying on synthetic datasets, this study utilizes point clouds from the Cranfield benchmark, incorporating CAD-sampled models and 3D scans of physical objects. The authors introduce real-world complexities such as noise and outliers, providing a more rigorous assessment. They evaluate three classical registration methods (i.e., GO-ICP, RANSAC, FGR) and three learning-based approaches (i.e., PointNetLK, RPMNet, ROPNet) using metrics such as recall, accuracy, computation time, and robustness to noise and partial data. This study can provide valuable findings that highlight the strengths and limitations of classical and learning-based registration techniques for real-world data, offering practical guidance for domain researchers in selecting methods based on accuracy, efficiency, and noise resilience.

Contribution 6 introduces a refined point cloud registration method that leverages geometric constraints and a dual-criteria evaluation process. It is noted that traditional point-to-point methods often suffer from inaccuracies due to erroneous matches and noise. The proposed approach enhances reliability by requiring only two correspondences (i.e., instead of the conventional three) to generate a transformation matrix, reducing computational complexity. Specifically, keypoints are detected to establish initial correspondences, with high-quality matches selected for improved alignment. Rotation and translation matrices are then computed using centroids and local reference frames. The optimal transformation matrix is determined based on the overlap ratio and inlier count. Experimental results demonstrate that integrating geometric constraints with a comprehensive evaluation strategy significantly enhances both accuracy and efficiency.

Contribution 7 presents an innovative LiDAR-based method for dynamic target detection, where motion states are evaluated by analyzing positional and geometric differences in point cloud clusters across consecutive frames. To accurately pair clusters representing the same target, a double registration algorithm is introduced, where a coarse registration is performed via iterative closest point (ICP) for initial pose estimation, followed by fine registration using random sample consensus and a four-parameter transformation for precise inter-frame alignment. This dual-step process standardizes coordinate systems, facilitating cluster association. Based on these paired clusters, the study constructs a classification feature system and employs the XGBoost decision tree for motion state evaluation. To improve training efficiency, a Spearman rank correlation-based bidirectional search reduces feature dimensionality, optimizing the classification subset. Meanwhile, a double Boyer–Moore voting–sliding window algorithm refines detection accuracy.

Contribution 8 introduces a robust solution for 3D object detection by effectively leveraging distance information and preserving critical point features, thereby enhancing the accuracy and reliability of scene understanding in complex environments. Specifically, the authors propose a set abstraction enhancement (SAE) network to address challenging issues raised by sparse and irregular point cloud data, which incorporates three key modules: an initial feature fusion module, a keypoint feature enhancement module, and a revised group aggregation module. By emphasizing distance information, the proposed network enhances the representation of distant objects, mitigating the decline in reflectivity that occurs with increased range. Moreover, it reinforces the intrinsic features of keypoints before they are combined with aggregated features, ensuring that essential semantic information is retained. Finally, the semantic coherence of the aggregated features improves the network’s ability to differentiate between objects. Experimental results demonstrate that the integration of distance features and the enhancement of keypoint characteristics contribute to the more accurate detection of objects at varying distances, addressing the challenges posed by point cloud sparsity and reflectivity attenuation.

Contribution 9 advances point cloud registration by addressing challenges in low-overlap environments, where traditional methods struggle due to their reliance on abundant, repeatable keypoints for accurate correspondence extraction. As a result, the authors propose a graph convolutional attention-based robust point cloud registration network (RRGA-Net) to optimize correspondences among sparse keypoints through a multi-layer channel sampling mechanism and a template matching module. By forming patches through feature weight filtering, the proposed network captures more comprehensive contextual features, which is crucial for effective registration in low-overlap scenarios. Moreover, the integration of self-attention mechanisms allows the network model to dynamically adjust weights based on relationships between points, enhancing the capture of both local and global features. Experimental results demonstrate that RRGA-Net exhibits robust performance, particularly excelling in low-overlap scenarios.

Contribution 10 presents a novel framework for accurately measuring cuboid and cylindrical objects using point cloud data from time-of-flight (ToF) sensors. ToF sensors often produce low-resolution, noisy data with self-occlusions and multipath interference, distorting object shape and size. To address these issues, the authors propose an enhanced superquadric fitting technique designed for noisy and incomplete point clouds. The proposed framework first performs ground plane rectification using fiducial markers to align the ground horizontally, followed by segmentation to isolate the related objects. A superquadric shape is then fitted using non-linear least squares regression. The proposed method is tested on objects of known dimensions placed on various surfaces, including aluminum foil, black/white posterboard, and black felt. Experimental results demonstrate that the enhanced superquadric fitting, particularly the bounding method, significantly improves accuracy, making this approach valuable for precise object measurement applications.

## 3. Conclusions

The evolution of point cloud acquisition and analysis has been propelled by AI advancements. This Special Issue compiles a diverse portfolio of contributions that address critical challenges in point cloud processing, sensing, and understanding. The selected studies push the boundaries of current knowledge by offering innovative solutions to existing challenges and unlocking new 3D applications. We anticipate that these developments can further expand the applications of point clouds across various industries, offering new opportunities and valuable insights for both researchers and practitioners to drive research and innovation in this field.
